# The use of pharmacogenetics to increase the safety of colorectal cancer patients treated with fluoropyrimidines

**DOI:** 10.20517/cdr.2019.04

**Published:** 2019-03-19

**Authors:** Elena De Mattia, Rossana Roncato, Chiara Dalle Fratte, Fabrizio Ecca, Giuseppe Toffoli, Erika Cecchin

**Affiliations:** Experimental and Clinical Pharmacology Unit, Centro di Riferimento Oncologico di Aviano (CRO) IRCCS, Aviano 33081, Italy.

**Keywords:** Fluoropyrimidines, pharmacogenetics, colorectal cancer, toxicity, *DPYD*, *TYMS*, *MTHFR*

## Abstract

Fluoropyrimidines (FP) are given in the combination treatment of the advanced disease or as monotherapy in the neo-adjuvant and adjuvant treatment of colorectal cancerand other solid tumors including breast, head and neck and gastric cancer. FP present a narrow therapeutic index with 10 to 26% of patients experiencing acute severe or life-threatening toxicity. With the high number of patients receiving FP-based therapies, and the significant effects of toxicities on their quality of life, the prevention of FP-related adverse events is of major clinical interest. Host genetic variants in the rate limiting enzyme dihydropyrimidine dehydrogenase (*DPYD*) gene are related to the occurrence of extremely severe, early onset toxicity in FP treated patients. The pre-treatment diagnostic test of 4 *DPYD* genetic polymorphisms is suggested by the currently available pharmacogenetic guidelines. Several prospective implementation projects are ongoing to support the introduction of up-front genotyping of the patients in clinical practice. Multiple pharmacogenetic studies tried to assess the predictive role of other polymorphisms in genes involved in the FP pharmacokinetics/pharmacodynamic pathways, *TYMS* and *MTHFR*, but no additional clinically validated genetic markers of toxicity are available to date. The development of next-generation sequencing platforms opens new possibilities to highlight previously unreported genetic markers. Moreover, the investigation of the genetic variation in the patients immunological system, a pivotal target in cancer treatment, could bring notable advances in the field. This review will describe the most recent literature on the use of pharmacogenetics to increase the safety of a treatment based on FP administration in colorectal cancer patients.

## Introduction

The development of adverse drug reactions related to a pharmacological treatment is a pivotal phenomenon burdening patients quality of life as well as the national health systems for the related economical expenses. Adverse drug reactions were reported to account for about 5% of all the hospital admissions in the UK in 2004^[1]^ with an economic burden of about half a billion of pounds every year. About a 0.3% rate of adverse drug reactions were estimated to lead to patients death accounting for 100,000 death annually in the US^[2]^, similarly to the UK^[1]^. Other Europe-based studies reported an estimate of 1,058 billion Euros per year as the estimated economic burden related to the hospital costs required to manage adverse drug reaction in Germany^[3]^; and 21 million dollars per year every 100,000 adult inhabitants in Sweden^[4]^**.**

When the attention is focused on the adverse drug reactions related to cancer therapy, these numbers dramatically increase. A revision of the adverse events occurred after one drug administration in more than 4,000 colorectal cancer (CRC) patients treated in the US in 2016 pointed out that more than 90% of the patients developed at least one toxic event of any grade of severity with a significant economic burden^[5]^.

The backbone of the pharmacological treatment of CRC patients is still represented by fluoropyrimidines (FP) [i.e., 5-fluorouracil (5-FU) and capecitabine], that have been used for more than 50 years in several combination regimens (mainly for the treatment of the advanced disease) or in monotherapy (in neo-adjuvant and early tumor stage adjuvant patients) in all the disease stages. FP are used not only in the treatment of CRC but also in a wide range of solid tumors including breast, head and neck and gastric cancer. Adverse drug reactions related to the use of FP are quite common, becoming very severe or even life-threatening in 10%-26% of treated patients^[6,7]^. In a significant minority (3%-5%) of patients, early, severe side effects occur even with conventional moderate doses of the drug^[8]^. The most frequently reported severe adverse events linked to the FP administration, both for the metastatic disease and in the adjuvant setting, are hematologic (neutropenia in the 40%-56% of patients) and gastrointestinal (diarrhea in the 10%-15% of patients). Occurrence of such events can lead to a dose reduction, treatment delay and/or therapy suspension potentially compromising the therapy efficacy^[9]^. We have recently revised the toxicity data of 743 CRC patients enrolled in prospective pharmacogenetic studies in our center (Experimental and Clinical Unit of CRO Aviano), all treated with FP-based regimens. The revision pointed out that 15.7% (95 patients) developed a severe to lethal toxicity (grade 3 to 5 according to NCI-CTC *vs*. 3.0). The large majority of these patients (55.7%) were treated in neo-adjuvant or adjuvant setting and 3 of them died due to toxicity. Moreover, the 23.3% of the study population (178 patients) developed at least one event of dose-limiting-toxicity (DLT, grade 3 or higher non-hematological or grade 4 or higher hematological toxicity) during therapy. The most common DLTs were non-hematological ones (154 patients), mostly represented by diarrhea, vomiting and hand-foot syndrome (HFS). On the other hand, the 38 patients reporting grade 4 or higher hematological toxicities suffered from neutropenia in the majority of cases^[10]^.

In this review the issue of patients genetic profiling to identify in advance CRC patients at risk of FP-related toxicity will be addressed.

## Pharmacogenomic guidelines and their implementation in the clinical practice

The debate on the opportunity to implement pharmacogenetic variants screening in the clinical practice is still ongoing and specifically the use of dihydropyrimidine dehydrogenase (DPD, encoded by *DPYD*) testing to spare FP-related toxic events occurrence is not yet mandatory. Particularly, the publication of European Society for Medical Oncology guidelines for the management of CRC patients^[11]^, not recommending an upfront *DPYD* genotyping for FP administration recently rekindled the debate^[12,13]^.

Up to date, even though more than 160 single-nucleotide polymorphisms (SNPs) have been reported in *DPYD*, only four of them presented a sufficient evidence of a clinical impact to be included in the current guidelines. The most dysfunctional variants known for their role in impairing DPD activity are DPYD*2A (rs3918290) and DPYD*13 (rs55886062) associated with an almost complete protein deficiency in homozygous individuals. C.2846A>T (rs67376798) and c.1236G>A (*DPYD*-Hap-B3; rs56038477) are instead related to a moderate loss of protein function. A number of studies described the impact of these SNPs on the outcome of an FP-based treatment in term of risk of developing severe to life-threatening toxicity both in large randomized clinical trials^[14,15]^, and in retrospective collection of unselected patients from the current clinical practice^[16]^. More recently, the pre-emptive genotyping of DPYD*2A, coupled with a front-line dose reduction was demonstrated to be feasible and effective in preventing toxicity occurrence^[17]^. In an effort to provide a global DPD metabolizing status for each patients and to provide personalized dosing guidelines based on the patients genotype for the four variants, a gene activity score was developed, taking into account the diplotype allelic combination of the variants^[18]^. Based on the single allele score, a patient’s specific estimation of the protein functionality is obtained, conferring to the patients a gene activity score ranging from 0 (completely dysfunctional) to 2 (completely functional), allowing to tailor the FP starting dose based on the patients genotype. The most recent version of shared pharmacogenetic guidelines are based on the definition of the patient gene activity score^[19]^.

Our group demonstrated in a large population of 763 CRC patients treated with a chemotherapeutic regimen including FP in whichever setting of treatment, that carriers of at least one variant in the four *DPYD* SNP panel were significantly more exposed to the risk of both acute (within the first three cycles) and chronic clinically relevant toxicity, defined as grade 3 or higher non hematological or grade 4 or higher hematological^[10]^. When stratifying the patients according to their *DPYD* activity score the trend for the toxicity risk was significantly increasing with the decrease of the patients activity score. The significant relationship between *DPYD* genotype and toxicity risk has been confirmed also when FP-based chemotherapy is given in combination with radiotherapy in more than 800 patients, confirming that even in this specific clinical setting an upfront dose adjustment based on *DPYD* genetic profile would be beneficial for patients safety^[20]^.

**Figure 1:**
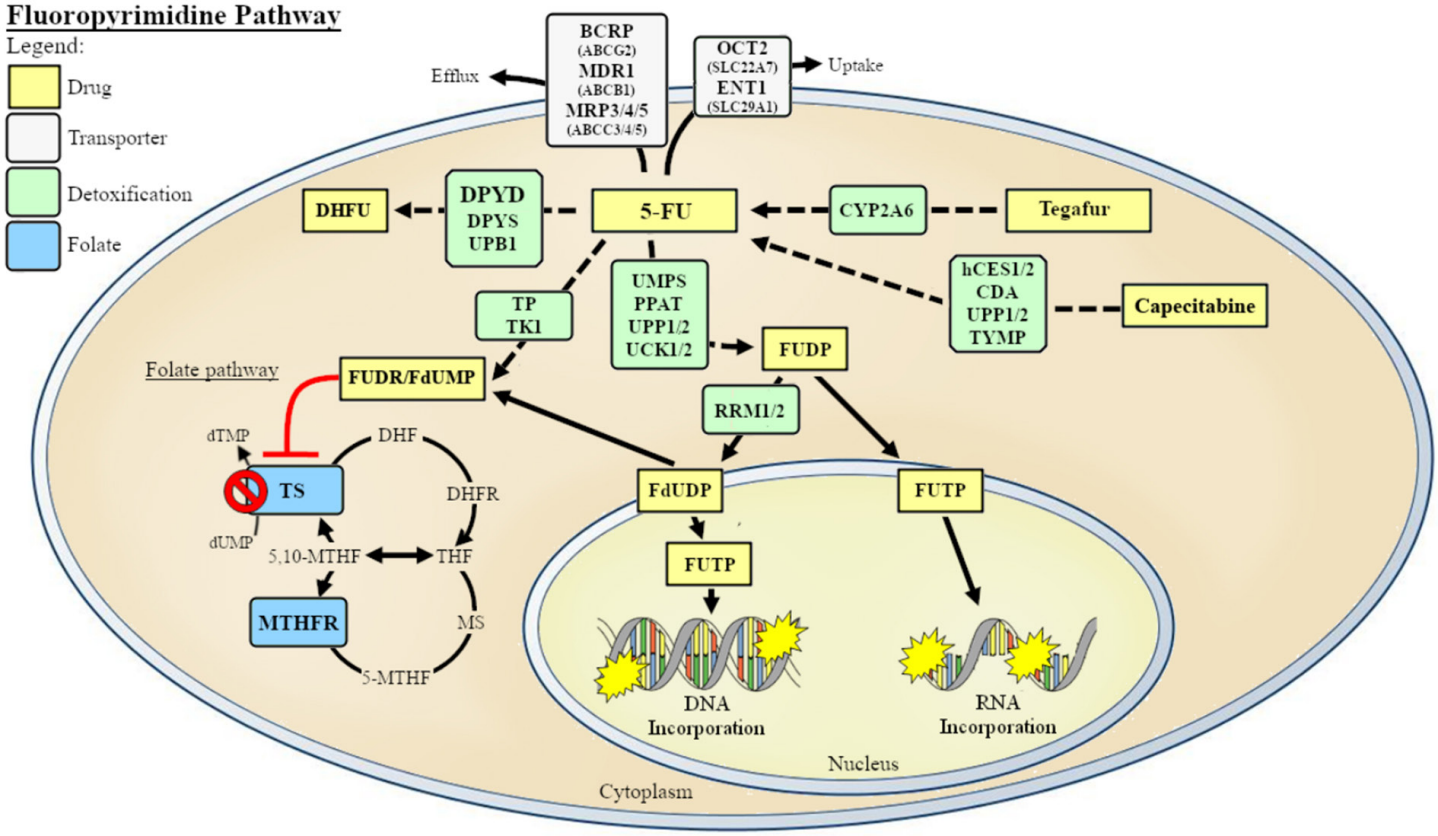
Metabolic pathway of fluoropyrimidines. 5-Fluorouracil (5-FU), is metabolized intracellularly to its active form 5-fluoro-2-deoxyuridine-5’-monophospate (5-FdUMP) through two consecutive reactions catalyzed by thymidine phosphorylase (TP) and thymidine kinase (TK). Another important metabolic enzyme is the ribonucleotide reductase, composed of large subunit RRM-1 and small subunit RRM-2, that converts fluorouridine diphosphate (FUDP) to fluorodeoxyuridine diphosphate (FdUDP), which preferentially affects DNA metabolism. 5-FU carries on his cytotoxic effect by mediating the formation of an inhibitory ternary complex, involving its metabolite 5-FdUMP, thymidylate synthase (TS, *TYMS*) and 5,10-methylentetrahydrofolate (5,10-MTHF). The formation of this complex inhibits TS activity, with subsequent diminution of thymidylate levels and consequent suppression of DNA synthesis. Dihydropyrimidine dehydrogenase (DPD, *DPYD*) is the first and rate-limiting enzyme of the fluoropyrimidines catabolic pathway converting 5-FU to dihydrofluorouracil (DHFU) while 5,10-methylenetetrahydrofolate reductase (MTHFR) catalyzes the irreversible conversion of 5,10-MTHF, required for DNA synthesis, to 5-MTHF, the primary methyl donor indispensable for nucleic acid methylation. Human carboxylesterase isoforms 1 and 2 (hCES1/2) and cytidine deaminases (CDAs) are necessary for capecitabine activation and metabolism. *UMPS* encodes the enzyme orotate phosphoribosyltransferase (OPRT), which catalyzes the conversion of 5-FU into fluorouridine monophosphate (FUMP), a common substrate for the production of cytotoxic metabolites that target RNA and DNA. Some ATP-binding cassette (ABC) and solute carrier (SLC) membrane transporter are involved in drug translocation of the drug

Available pharmacogenetic guidelines for FP are currently based on the screening of four genetic variants in the *DPYD* gene (www.pharmgkb.org). DPD is the first and rate-limiting enzyme of FP catabolic pathway [Figure 1]. A recent publication reported the results of a prospective clinical trial testing the utility and feasibility to apply current pharmacogenetic guidelines in the clinical practice to patients treated with an FP-based therapy^[19]^. The trial enrolled more than 1,100 cancer patients and demonstrated that an up-front adjustment of FP dosage based on the genotype of the patients contributed to expose the patients to a more homogeneous drug plasma level. This resulted in a similar risk to develop severe toxicity between risky variants carriers and non-carriers. The risk to develop severe toxic side effects in poor metabolizer patients was decreased in comparison to historical cohorts of patients treated according to the standard practice^[21]^.

A clinical implementation experience of the *DPYD* four relevant SNPs typing prior to FP treatment has been reported with a positive effect on patients outcome and with a successful up-take by the treating oncologists^[22]^. However this is not the common practice in the majority of the Health Care Systems, and the clinical utility of the test is far from being widely acknowledged^[23]^. One of the major concerns is the lack of formal health technology assessment studies, including cost-effectiveness and cost-consequences evaluation.

We previously reported that the costs required to manage toxicity related to chemotherapy are associated to the patient’s genotype for specific pharmacogenetic toxicity risk variants^[24]^. Last year we performed a cost-analysis in a large group of Italian CRC patients treated with FP-based chemotherapy demonstrating that carriers of the four *DPYD* variants have higher toxicity management costs than non-carriers (carriers €2,972; 95% confidence interval [(CI), €2,456-€3,505 *vs*. noncarriers €825; 95%CI, €785-€864] and that the mean toxicity management cost per patient is related to the patients *DPYD* gene activity score. In a subgroup of patients treated with an association of FP and irinotecan, the incremental cost between carriers and non-carriers increased further (in the amount of €2,975), by including the UGT1A1*28 genotype^[25]^. Another European group prospectively demonstrated that upfront genotyping of DPYD*2A is feasible, improves safety of FP therapy for patients, and is more likely cost saving^[17]^. More recently a large prospective trial, from the same group, provided evidence of the cost-effectiveness of an upfront *DPYD*-guided dose individualization. The *DPYD* screening strategy resulted in a net cost saving of 51€ compared with the non-screening one^[26]^.

Probably the ultimate proof to support the introduction of pharmacogenetics in the clinical practice will derive from the several ongoing implementation projects in Europe and in the US^[27]^. Specifically, the only ongoing project in Europe since the 1st of January of 2016 is Ubiquitous Pharmacogenomics (U-PGx) (www.upgx.eu)^[28,29]^. U-PGx will enroll, genotype, treat, and follow up at least 8,000 patients in Europe, with the financial support of Horizon 2020 granting program, under the coordination of Leiden University Medical Center. It will test, within a large randomized clinical trial conducted in 7 different health care European contexts, the clinical validity and utility of the pharmacogenomics approach in the clinical practice for a list of 43 drugs with a pharmacogenetic guideline available, including FP-*DPYD* gene drug interaction.

## Pharmacogenetic exploratory markers of fluoropyrimidines

5-FU, is a pyrimidine analog, belonging to the antimetabolites category, and acts through its active metabolite 5-fluoro-2-deoxyuridine-5’-monophospate that is a nearly irreversible inhibitor of thymidylate synthase (TS, encoded by *TYMS*). A comprehensive overview of FP-related pathways is depicted in Figure 1.

Beside the catabolic enzyme DPD, other important pharmacogenes with an impact on the risk to develop FP related toxicity, belong to the folate metabolism pathway^[30]^. An alteration in the functionality of the proteins involved in this pathway could modulate the activity of FP and consequently the drug toxicity profile. Particularly, variations in genes encoding TS and 5,10-methylenetetrahydrofolate reductase (MTHFR) represent the most investigated markers^[31,32]^. More recently other FP related genes as well as specific immuno-related genetic profiles have been considered as potential markers of adverse drug reaction occurrence. The following paper sections will cover the most recent literature regarding the emerging pharmacogenetic data related to the development of adverse drug reactions in CRC patients treated with FP.

### Dihydropyrimidine dehydrogenase

As already mentioned, DPD is the first and rate-limiting enzyme in the FP catabolic pathway converting 5-FU to dihydrofluorouracil; the activity of this protein is shown to be highly variable among individuals spanning from partial (about 3%-5% of the entire population) to complete loss of the enzyme functionality (about 0.2%-0.3% of the entire population)^[33]^. DPD deficiency is demonstrated to be partly linked to some genetic polymorphisms and to be responsible of life-threatening early toxic events that occur in about 0.5% of patients receiving 5-FU^[33,34]^. DPYD*2A (rs3918290) DPYD*13 (rs55886062), c.2846A>T (rs67376798, D949V) and c.1236G>A-HapB3 (rs56038477), the genetic variants included in the international pharmacogenetic guidelines for drug adjustments do not cover all the cases of DPD deficiency and their screening could not predict with sufficient sensitivity the risk of early severe toxic events induced by FPs. The *DPYD* gene is highly polymorphic and other genetic variants are likely to impact the enzyme activity and the risk to develop severe toxicity. Some additional *DPYD* variants (e.g., rs75017182, rs1801158 rs2297595, rs17376848, rs72549309, rs1801265, rs1801160) have been suggested to be clinically relevant predictor of FPs associated toxicity and could be considered, alone or in score combination, for improving available dosing guidelines [Table 1]^[14,18,21,22,33,35-37]^. However at present robust evidence has been generated only for the DPYD*6 (rs1801160) variant^[14,37,38]^*.* Ruzzo *et al*.^[38]^ highlighted that DPYD*6, together with *DPYD* rs2297595 and DPYD*2A, were not only linked to a higher risk of toxicity but also to the time-to-toxicity parameter, emphasizing the acute occurrence of genetically determined toxicity occurrence. The introduction of the dimension of time allows a better characterization of the gene-linked toxicity profile and is particularly sensitive in the case of few observations (i.e., rarity of some genotypes) and genetic variants with moderate functional effects. Moreover, the application of the recently developed high-throughput next generation sequencing (NGS) technology to samples obtained from CRC patients exhibiting extreme toxicity phenotypes, will allow to investigate and possibly identify additional novel and rare variants, significantly impacting the DPD activity, that could be integrated into the pharmacogenetic algorithm to further improve the FPs administration^[18,36,39]^.

**Table 1:** Relevant *DPYD* Allele functionality table

DPYD Haplotype	rsID	Nucleotide change^a^	Protein change^b^	Allele Functional Status	Activity Score	Evidence supporting function	Ref.
*2A	rs3918290	c.1905+1G>A	N/A	No function	0	Strong^c^	[40]
*5	rs1801159	c.1627A>G	p.I543V	Normal	1	Strong	[40]
*9A	rs1801265	c.85T>C	p.C29R	Normal	1	Strong	[41]
*13	rs55886062	c.1679T>G	p.I560S	No function	0	Strong	[40]
	rs67376798	c.2846A>T	p.D949V	Decreased	0,5	Strong	[42]
*HapB3*	rs75017182, rs56038477, rs56276561	c.1129-5923C>G, c.1236G>A, c.483+18G>A	N/A, p.E412E, N/A	Decreased	0,5	Strong	[43]
*4	rs1801158	c.1601G>A	p.S534N	Normal	1	Moderate^d^	[40]
*6	rs1801160	c.2194G>A	p.V732I	Normal	1	Moderate	[40]
*7	rs72549309	c.295_298delTCAT	p.F100Sfs	No function	0	Moderate	[42]
	rs2297595	c.496A>G	p.M166V	Normal	1	Moderate	[42]
	rs17376848	c.1896T>C	p.F632F	Normal	1	Moderate	[40]

^a^Nucleotide changes according to reference sequence NM_000110.3; ^b^protein changes according to reference sequence NP_000101.2; ^c^strong evidence supporting function (from both in vitro and clinical studies); ^d^moderate evidence supporting function (from *in vitro* and clinical/*ex vivo* studies). Adapted from Amstutz *et al.*^[19]^ (updated table on 25/05/2017)

The combination of the *DPYD* genetic variability with the measurement of the DPD enzymatic activity is probably the most effective strategy to identify patients at risk of severe and potentially fatal FPs-associated toxicityand has been successfully integrated in the clinical practice improving patients outcome^[44,45]^. Some Dutch and French studies have recently demonstrated how Uracil levels could be used successfully to anticipate severe toxicities^[46,47]^. A recent study demonstrated that an algorithm, integrating information of high-throughput *DPYD* genotyping with the determination of DPD enzyme phenotype, could effectively identify DPD deficient individuals, and is suitable for routine clinical application^[33]^. A score predicting the risk for severe toxicity based on *DYPD* mutation and 5-FU degradation rate among other clinic-pathological features (i.e., age, number of drugs administered) was also developed and reported to be an easy and low-cost method fitting the requirements for clinical use^[48]^.

In addition to the *DPYD* genetic profiling, also the epigenetic control of the gene expression has been demonstrated to have a regulatory effect on DPD, and consequently a potential role in determining the likelihood to experience severe FPs-related toxicity. Particularly, promising data are available on the association of rs895819 in the gene encoding for *miR-27a*, responsible for *DYPD* gene silencing with early-onset FPs toxicity. The SNP was reported to improve toxicity risk stratification in patients that are carriers of the known *DPYD* risk polymorphisms^[49]^.

### Thymidylate synthase

An altered intracellular expression of TS could significantly modify the cell sensitivity to drug and consequently its therapeutic effects; in particular a high TS level seems to contribute to diminishing the cytotoxic activity of FPs^[50,51]^. Three polymorphisms (rs45445694, rs2853542, rs16430), in moderate linkage disequilibrium (LD), located in the untranslated regions (UTRs) and associated with a change in gene expression, TS mRNA stability and/or TS levels, are the most studied *TYMS* variants as potential pharmacogenetic markers of toxicity risk^[52,53]^. TS promoter-enhancer region (TSER) polymorphism (*TYMS* 2R/3R repeat, rs45445694), in the 5’UTR, is characterized by a variable number of tandem repeats of a 28 base pairs sequence giving rise primarily to alleles of two (2R) and three (3R) repeats; an higher number of repeats (i.e., 4R) was also described. An higher number of repeats (i.e., 3R or higher) was demonstrated to be associated with a higher TS expression level. More recently, additional functional variant within the 5’-UTR region has been identified, consisting of a G to C substitution at the 12th nucleotide of the second repeat of the 3R allele (TSER 3R G/C, rs2853542). The 3RC/3RC genotype was reported to cause a lower transcriptional activity of TS, comparable with the 2R/2R genotype, whereas the presence of 3RG is correlated with higher transcriptional activity respect other genotypes. The third variant consists in a 6-bp deletion at position 1,494, within the 3’UTR (6bp+/6bp-, rs34489327), and was shown to decrease RNA stability, and thereby to negatively influence TYMS mRNA and TS protein expression *in vitro*. It has been reported that TSER and TS-3’UTR polymorphisms are in linkage disequilibrium^[41]^.

The large number of available studies was integrated by two meta-analyses aimed at clarifying the real impact of these *TYMS* variants in modulating FPs toxicity^[39,54]^. The first^[54]^, focusing only on *TYMS* 5’UTR rs45445694 variant, and pooling 2,402 subjects, of the most part Europeans, receiving FPs based-therapy, demonstrated that the *TYMS* genotype associated with the lowest protein expression (2R/2R) was significantly correlated with an increased risk of adverse events. This result was limited by substantial heterogeneity of the studies included in the meta-analysis. However, the data were confirmed in a sensitivity analyses performed in order to consider potential confounding factors (i.e., ethnicity, chemotherapeutic regimen, biological sample used for DNA extraction, and the method of genotyping).

It should be also noticed that the predictive effect size of *TYMS* markers on toxicity risk, even if significant, is very small and therefore translates into a limited clinical utility. The second recent systematic meta-analysis^[39]^, including 4,855 CRC Caucasian patients receiving 5-FU or capecitabine both alone or in combinatorial regimens, evaluated the predictive role on toxicity risk of *TYMS* 5’UTR and 3’UTR genetic variants. This study reported that *TYMS* 5’UTR-2R and *TYMS* 3’UTR-6bp ins alleles were associated with an increased risk of severe toxicity after infusional or bolus 5-FU monotherapy. These two markers were found to be predictive of severe toxicity also in patients treated with capecitabine. A score, based on the number (0 to 4) of *TYMS* toxicity risk alleles, was further created and was shown to be a good predictor of any capecitabine associated toxicity with a weaker evidence of association in patients receiving infusional or bolus 5-FU monotherapy. When FP combination therapy was considered, none of the *TYMS* polymorphisms were associated with toxicity risk, probably due to the reduced FP dosage in these regimens, the presence of overlapping confounding toxicities, or the non-adequate sample size of the studies performed. Globally, even if *TYMS* polymorphisms could be predictive of toxicity in FPs monotherapy, especially when capecitabine is administered, the relatively weak size effect and the lack of association in the most commonly used combinatorial regimes, require further evidence to support their use in clinical practice.

This is consistent with the results of an umbrella systematic review concluding that *TYMS* polymorphisms had a statistically significant relationship with FPs-induced toxicity but that this relationship was relatively weak and without any clinical significance^[55]^. Therefore, *TYMS* variants could possibly be incorporated into a panel of other predictive markers of FPs toxicity and a personalized drug dosing based on *TYMS* genotyping could be applied only after further exhaustive investigation of the effect of a pre-emptive dose reduction on the patient outcome.

However, even if the impact of *TYMS* variants on FPs-related toxicity risk appears to be minimal, their effect in determining the FPs-based therapy effectiveness is more fully established. A recent randomized trial (MAGIC trial) with a control group, has found that patients treated with 5-FU containing regimen and harboring the *TYMS* genotype 2R/2R, associated with the low expression phenotype, present a superior OS respect those with 2R/3R or 3R/3R genotype; this difference was not detected among patients treated with surgery alone suggesting a specific interaction between *TYMS* genetic status and treatment. Patients with the *TYMS* 2R/2R genotype have showed also a tendency towards an higher rate of response to FP chemotherapy compared to those with the 2R/3R or 3R/3R genotype^[56]^. Accordingly, in another pooled analysis of three prospective clinical studies the *TYMS* genotype 3R/3R genotype was associated with an inferior objective response (ORR) rate to FPs-containing regime; considering also the G to C substitution in the 3R allele, patients carrying the 3RG/3RG genotype appeared to have the lowest ORR^[49]^. Interestingly, a phase II study further demonstrated that the prospective use of *TYMS* genotyping could direct the neoadjuvant 5-FU-based chemoradiotherapy (CRT) in patient affected by rectal cancer^[57]^. Particularly, patients carrying the T*YMS* 2R/2R or 2R/3R genotypes, who were supposed to have a favorable response to 5-FU, were treated with the standard CRT, while patients with the T*YMS* 3R/3R or 3R/4R genotypes, who were deemed to not derive a significant benefit from 5-FU, were treated with an alternative regimen. These findings support the feasibility of a personalized treatment aiming to improve the therapy effectiveness based on TYMS genotyping and encourage further evaluation of this genotype-based strategy.

**Figure 2:**
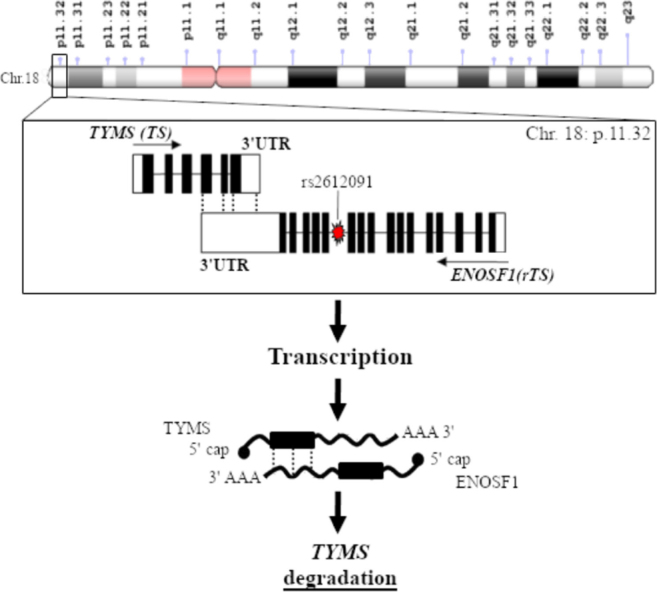
Regulation of *TYMS* expression by its antisense mRNA, enolase superfamily member 1 (*ENOSF1*). *ENOSF1* gene, encoding a mitochondrial enzyme also known as reverse TS (rTS), is located adjacent to *TYMS* and a regulatory interaction between the two genes/proteins has been suggested. High expression level of ENOSF1 has been found to suppress the TS expression level by either inducing the production of an antisense RNA targeting *TYMS* mRNA or by inducing the expression of a protein (i.e., rTS-β) modulating the TS protein level at the post-transcriptional phase. *ENOSF1* polymorphisms (i.e., rs2612091) could perturb this TYMS mRNA-antisense mRNA autoregulatory complex

Interesting data has been also published on variants in enolase superfamily member 1 (*ENOSF1*) gene encoding a mitochondrial enzyme also known as reverse TS (rTS). A work on 968 Caucasian capecitabine-treated patients reported that the common G-allele of rs2612091 variant, which lies within an intron of *ENOSF1* downstream of *TYMS*, was associated with increased global grade ≥ 3 toxicity and HFS^[58]^. A more detailed analysis further showed that the rs2612091 signal appears to fully explain the previously reported associations between 5-FU/capecitabine toxicity and the *TYMS* polymorphisms (5’UTR 2R/3R and 3’UTR 1494del6 +/-). These surprising results imply that the *TYMS* 5’UTR and 3’UTR toxicity association signals derive from LD between these *TYMS* polymorphisms and the *ENOSF1* tagging polymorphisms (TagSNP) rs2612091; suggesting that the toxicity polymorphisms in the *TYMS* region may actually act through ENOSF1^[58]^. Regarding the molecular mechanism underlying these observations, the *ENOSF1* rs2612091 variant was reported to affect *ENOSF1* mRNA expression. High expression level of *ENOSF1* has been found to suppress the TS expression level by either inducing the production of an antisense RNA targeting *TYMS* mRNA or by inducing the expression of a protein (i.e., rTS-β) modulating the TS protein level at the post-transcriptional phase [Figure 2]. Hence a major role of ENOSF1 on the cell’s sensitivity to the cytotoxic effects of FPs, as compared to TYMS could be speculated^[49]^. The role of *ENOSF1* rs2612091 as candidate marker of FPs-related toxicity was further confirmed by the study of García-González *et al*.^[59]^ on 239 Caucasian CRC patients reporting an association between this variant and grade > 1 HFS. An effect of the rs2612091 variant on OS of patients receiving capecitabine-containing regimen has been also reported^[49]^, emphasizing a potential clinical role of this marker.

### 5,10-methylenetetrahydrofolate reductase

MTHFR, is another key enzyme for intracellular folate homeostasis and metabolism, catalyzing the irreversible conversion of 5,10-methylentetrahydrofolate, required for DNA synthesis, to 5-methyltetrahydrofolate, the primary methyl donor indispensable for nucleic acid methylation [Figure 1]^[31,32,60,61]^. Two non-synonymous *MTHFR* variants, rs1801133 and rs1801131, are among the most studied genetic markers for identifying predictors of FPs-related severe toxicity. These polymorphisms, in high LD, were associated with a decreased enzyme activity, an increased level of homocysteine and an altered distribution of folate, with a synergic effect on the haplotype arrangement^[31,62]^. It could be supposed that a reduced MTHFR functionality due to the polymorphic variants might lead to an increased 5,10-methyleneTHF concentration that, enhancing the formation and stability of the ternary inhibitory complex, might increase the cytotoxic effect of 5-FU or capecitabine and therefore the rate of adverse effects. This hypothesis was tested by some pharmacogenetic studies, though without generating the robust and validated evidences necessary for supporting the use of rs1801133 and rs1801131 markers in the clinical practice. Particularly, three meta-analyses^[39,54,55]^ were performed integrating the data derived from published studies, that present mainly a small cohort size, in order to clarify the role of *MTHFR* polymorphisms in predicting toxicity risk in CRC patients receiving FPs. Globally, *MTHFR* rs1801133 and rs1801131 variants were not found to have any association with FPs-induced toxicity, with an exception of a small protective effect of rs1801133 on the risk of neutropenia after bolus 5-FU monotherapy in Caucasian patients. However, the more recent study by Pellicer *et al*.^[63]^ has revived the interest about a potential role of *MTHFR* polymorphisms as predictors of FPs-related toxicity. In fact, this study using a novel approach based on exome sequencing of samples derived from CRC patients exhibiting extreme toxicity phenotypes, and a subsequent validation of the most promising markers on a large set of capecitabine-treated patients, has reported that*MTHFR* rs1801133, among other genetic markers, is still one of the most significantly associated with the delayed administration of chemotherapy due to toxicity. In another recent comprehensive analysis of genetic variants in folate-mediated one-carbon pathway on more than one thousand CRC patients receiving 5-FU-containing regimes, the same *MTHFR* rs1801133 polymorphism has been also reported to be associated with OS^[64]^. These data claim further investigations with an adequate statistical power and a homogeneous cohort of patients and treatment modality to definitively clarify the utility of*MTHFR* polymorphisms as parameters for toxicity risk stratification.

### Other pharmacogenes

Preliminary results highlighted a potential role of the genetic variants in thymidine phosphorylase (*TYMP*) and uridine monophosphate synthase (*UMPS*), related to the folate homeostasis; *ABCB1* and *SLC22A7* involved in the drug transmembrane translocation; and cytidine deaminase (*CDA*) enzyme necessary for capecitabine activation, as toxicity markers in FP-treated patients^[39,59,63,65,66]^. Also variants in carboxylesterase 1 (*CES1)* and *CES2*, required for capecitabine metabolism, were considered as FP pharmacogenetic markers, but without generating positive results^[39,58,63]^.

The protein product of *UMPS* gene is the orotate phosphoribosyltransferase enzyme, which catalyzes the conversion of 5-FU into fluorouridine monophosphate, a common substrate for the production of cytotoxic metabolites that target RNA and DNA. Studies on Japanese CRC patients receiving 5-FU as adjuvant chemotherapy reported that the functional missense variant rs1801019 in *UMPS* was associated with more severe symptoms of GI toxicity such as grade 3 diarrhea and grade 2-3 anorexia^[65,66]^. However, the predictive role of this *UMPS* marker on GI side effects was not reproduced in a group of Caucasian CRC patients^[67]^.

The work of García-González *et al*.^[59]^ evidenced a potential predictive role on capecitabine-related toxicity also for other genetic markers. Particularly, the ABCB1*1 haplotype (rs1128503-C, rs2032582-G, and rs1045642-C) was associated with a high risk of grade > 2 diarrhea and overall toxicity (any adverse drug reactions classed as grade 3 or higher) as well as with a delay in drug administration or capecitabine dose reduction, while the *CDA* rs2072671-A was associated with a high risk of overall toxicity. A score based on *ABCB1-CDA* polymorphisms was also developed and showed to efficiently predict patients at high risk of severe overall toxicity after capecitabine administration. The role of *ABCB1* markers as predictors of the inter-individual variability in the capecitabine toxicity profile is a novel finding that should be further investigated. On the contrary the involvement of *CDA* rs2072671variant in determining the FPs related adverse reactions in more controversial since the existence of discrepant results^[39,68]^ probably due to differences in performed studies (study design, sample size, criteria severe toxicity cut-off establishment, concomitant medication). Two other functionally-relevant *CDA* polymorphisms in the promoter region, rs532545 and rs602950 variants, were associated with an increased risk to develop severe toxicity, and specifically grade 2-4 diarrhea and grade ≥ 3 HFS, by a studies on a large cohort of capecitabine-treated patients^[69,70]^. Another polymorphism, the rs3215400 that corresponds to a promoter insertion associated with enhanced CDA expression, in combination with a CDA ultra rapid phenotype, was suggested to contribute to a toxic deathdespite being *DPYD* and *TYMS* wild-type status in a patient treated with capecitabine^[71]^.

Hence, variants in *CDA* gene represent a good candidate targets for future pharmacogenetic investigation aiming at determining whether testing for these variants is clinically useful.

An additional investigated marker in the folate pathway is the TYMP enzyme and the related genetic variants. However, most of the studies failed to find an association between *TYMP* polymorphisms and capecitabine-related adverse events^[39]^. Only an investigation on 254 Caucasian CRC patients treated with 5-FU or capecitabine reported that the missense *TYMP* rs11479 variant was associated with FP dose modifications and/or severe adverse events. However a further analysis evaluating the potential value of testing the combined *TYMP* rs11479 and *DYPD* mutations signature revealed a still poor clinical impact highlighting the need to discover further novel genetic markers that could improve the predictive algorithm^[68]^.

A multi gene approach was attempted by the group of Pellicer *et al*.^[63]^, that investigated 23 TagSNP in FPs-pharmacodynamics genes on 301 Caucasian CRC patients receiving capecitabine-based chemotherapy, in order to find new genetic variants predicting individual risk of chemotherapy-induced severe adverse reactions. This study revealed ten polymorphisms associated with severe capecitabine toxicity: *CDA* rs1048977, rs12726436, and rs2072671; *DPYD* rs12119882*; TYMS* rs2853741*; TYMS/ENOSF1* rs699517; *SLC22A7* rs2270860 and rs4149178; *UMPS* rs2279199 and rs4678145. Except for rs2072671, all the observed associations were not previously reported, suggesting that the use of TagSNPs method could be a successful strategy to find new predictors of adverse reactions to capecitabine improving the power of currently available tests. All these promising candidate proteins involved in the FPs pathway should represent the target of future research aiming at discovering and validating additional biomarkers to be integrated in the pharmacogenetic test. A similar approach was adopted in a study conducted on a small group of CRC patients receiving triplet (irimotecan, 5-FU, and oxaliplatin) hepatic artery infusion plus intravenous cetuximab for unresectable liver metastases^[72]^. The analysis, conducted within the prospective European trial OPTILIV (NCT00852228) pointed out six ADME genes (i.e., *CYP2C9*, *CYP2E1*, *UGT1A6*, *SLCO1B3*, *SLC22A1*, and *ABCB1*) which polymorphisms were associated with the risk of developing severe toxicity. Patients in a phase III randomized clinical trial (NCT00486213) were also investigated in a genome-wide association study to find novel genetic determinants of capecitabine-related HFS^[73]^. A set of exploratory markers, including three novel *DPYD* variants, were significantly associated with grade 2 or higher HFS. If validated, these markers could further improve the management of patients receiving capecitabine. Another more recent work by Pellicer *et al*.^[37]^ already mentioned above, moved from a candidate SNP to an exome NGS approach to obtain a global profiling of the germline variability of genes involved in capecitabine transport, metabolism and mechanism of action. Beside confirming the pivotal role of *DPYD* and *MTHFR* polymorphism as predictors of FP-related toxicity, this study highlighted also some previously unreported genetic variantsas potential additional toxicity markers. These findings suggest the utility to adopt sequencing-based strategies to provide a broad pharmacogenetic overview and to expand the actual knowledge about genetic markers of FPs-related toxicity.

### Immuno-related genes

Immune system has been extensively investigated for its role in cancer treatment, and some data suggest that it could also contribute to determine the severity of drug related toxicity, including FPs. The immune system has been reported to play a key role in the pathogenesis of the gastrointestinal toxicity subsequent to FPs administration. In particular, inflammatory mediators such as tumor necrosis factor-α and interleukin 1 beta, seemed to be implicated in the complex mechanism underlying the toxic damage to the GI epithelium (i.e., mucositis) induced by some chemotherapeutics including 5-FU^[15]^. In this context, we recently published discovery/replication study on more than 400 mCRC patients treated with a first-line 5-FU containing regimen adopting a TagSNP approach to evaluate the systemic variability of 22 inflammation-related genes. The study highlighted that a polymorphism in thesignal transducer and activator of transcription 3 (STAT-3) encoding gene (i.e., rs1053004), a transcriptional factor triggered in response to the binding of some pro-inflammatory, and in the vitamin D receptor (i.e., rs11574077), a nuclear receptor acting as inflammation sensor, were significantly associated with the risk of developing severe GI toxicity. These findings highlight the importance of the inflammatory response mediators in the pathobiology of drug-induced mucosal injury, a topic that requires more attention in further pharmacogenetic investigations^[74]^.

We have also reported in another study that polymorphisms (rs371194629, rs1610696) in the gene encoding the Human Leukocyte Antigen G (HLA-G), involved in the immune modulation, could contribute in explaining the inter-individual differences in toxicity profiles of CRC patients receiving FPs containing therapy^[75]^. *HLA* polymorphisms as well as variants in the gene encoding for the pro-inflammatory cytokine interferon-γ (*IFNG*-rs1861494)^[76,77]^ and KIR-HLA^[78]^ have been also reported to be associated with the recurrence risk and OS of stage II-III patients receiving 5-FU-containing adjuvant therapy further stressing the relevance of immunogenetic markers for treatment personalization. The involvement of the immune system and inflammation process in determining FPs-based therapy clinical outcome administration is surely a intriguing topic warranting further pharmacogenetic investigation.

## Conclusion

The role of germline variants in the prediction and management of drug-related adverse events in CRC has been a matter of widespread investigation. Despite the well-established clinical validity of some pharmacogenetic markers and the publication of shared guidelines for FP and irinotecan, their use in the clinical practice is still object of debate. We have herein reported already available pharmacogenomic guidelines with ongoing efforts to implement them in the clinical practice, and new exploratory markers on the way to clinical validation, to move forward the fields of pharmacogenomics and precision medicine.

Collectively, the current literature data indicate that the loss of function *DPYD* polymorphisms are the only pharmacogenetic markers that have been found to have clinical and statistical significance for the predication of FPs-induced toxicity. However, neither the National Comprehensive Cancer Network, nor the European Society of Medical Oncology currently recommend their use in the clinical practice reflecting the challenge to integrate pharmacogenomics in clinical practice. Several research projects are currently ongoing to bring the ultimate proofs of the clinical validity and utility of pharmacogenomics implementation to improve patients management and to increase drug safety. Several clinical implementation projects are on-going in Europe and in the US and the results of some genotype directed clinical trials have been published demonstrating the feasibility of this approach and its applicability in the clinical practice^[79-81]^.

Other genetic targets as *TYMS* and *MTHFR* genotypes have only a small role to play in this context. *TYMS* and *MTHFR* markers could probably have a feasible application when integrated in a polygenic model that combines the effect of the single variants^[82]^. As example a discovery/validation study conducted on 302 Caucasian Dukes’ stage B2 and C colon cancer patients homogeneously treated with 5-FU based regimen, demonstrated that a specific combination of *MTHFR* rs1801131 and *TYMS* 3’-UTR ins/del (rs16430) polymorphisms, instead the single locus, could represent a significant predictor of increased toxicity risk^[83]^.

A lot of research has been focused to define additional predictive biomarkers to increase the sensitivity of the available pharmacogenetic tests for FP. A significant contribution in this sense could be provided by the research on the rare genetic variants, what emerging role has been pointed out by the advancement of the NGS technologies. From a recent revision of the 1K genome project results it appeared that about 30 to 40% of the inter-individual variability in drugs ADME (adsorption, distribution, metabolism, and excretion) and nuclear receptors genes is related to rare genetic variants that are not commonly screened in the pharmacogenetic studies^[84]^ and can be responsible for the observed variability in term of drug toxicity and pharmacokinetics. It is likely that future research, based on new and more comprehensive genetic analysis strategies will highlight more complex and heterogeneous genetic profiles addressing the issue of unexplained severe toxic events occurrence.

## Declarations

### Copyright

© The Author(s) 2019.

## References

[1] Pirmohamed M, James S, Meakin S, Green C, Scott AK (2004). Adverse drug reactions as cause of admission to hospital: prospective analysis of 18 820 patients.. BMJ.

[2] Lazarou J, Pomeranz BH, Corey PN (1998). Incidence of adverse drug reactions in hospitalized patients: a meta-analysis of prospective studies.. JAMA.

[3] Rottenkolber D, Schmiedl S, Rottenkolber M, Farker K, Saljé K (2011). Adverse drug reactions in Germany: direct costs of internal medicine hospitalizations.. Pharmacoepidemiol Drug Saf.

[4] Gyllensten H, Hakkarainen KM, Hägg S, Carlsten A, Petzold M (2014). Economic impact of adverse drug events--a retrospective population-based cohort study of 4970 adults.. PLoS One.

[5] Latremouille-Viau D, Chang J, Guerin A, Shi S, Wang E (2017). The economic burden of common adverse events associated with metastatic colorectal cancer treatment in the United States.. J Med Econ.

[6] Meyerhardt JA, Mayer RJ (2005). Systemic therapy for colorectal cancer.. N Engl J Med.

[7] Twelves C, Wong A, Nowacki MP, Abt M, Burris H (2005). Capecitabine as adjuvant treatment for stage III colon cancer.. N Engl J Med.

[8] André T, Quinaux E, Louvet C, Colin P, Gamelin E (2007). Phase III study comparing a semimonthly with a monthly regimen of fluorouracil and leucovorin as adjuvant treatment for stage II and III colon cancer patients: final results of GERCOR C96.1.. J Clin Oncol.

[9] Sugihara K, Ohtsu A, Shimada Y, Mizunuma N, Gomi K (2012). Analysis of neurosensory adverse events induced by FOLFOX4 treatment in colorectal cancer patients: a comparison between two Asian studies and four Western studies.. Cancer Med.

[10] Chiara DF, Jerry P, Rossana R, Elena DM, Fabrizio E (2018). DPYD gene activity score (GAS) predicts dose-limiting toxicity in fluoropyrimidine-treated colorectal cancer patients.. JMCM.

[11] Van Cutsem E, Cervantes A, Nordlinger B, Arnold D, ESMO Guidelines Working Group (2014). Metastatic colorectal cancer: ESMO clinical practice guidelines for diagnosis, treatment and follow-up.. Ann Oncol.

[12] Danesi R, Del Re M, Ciccolini J, Schellens JHM, Schwab M (2017). Prevention of fluoropyrimidine toxicity: do we still have to try our patient’s luck?. Ann Oncol.

[13] Deenen MJ, Meulendijks D (2017). Recommendation on testing for dihydropyrimidine dehydrogenase deficiency in the ESMO consensus guidelines for the management of patients with metastatic colorectal cancer.. Ann Oncol.

[14] Boige V, Vincent M, Alexandre P, Tejpar S, Landolfi S (2016). DPYD genotyping to predict adverse events following treatment with flourouracil-based adjuvant chemotherapy in patients with stage III colon cancer: a secondary analysis of the PETACC-8 randomized clinical trial.. JAMA Oncol.

[15] Lee AM, Shi Q, Pavey E, Alberts SR, Sargent DJ (2014). DPYD variants as predictors of 5-fluorouracil toxicity in adjuvant colon cancer treatment (NCCTG N0147).. J Natl Cancer Inst.

[16] Toffoli G, Giodini L, Buonadonna A, Berretta M, De Paoli A (2015). Clinical validity of a DPYD-based pharmacogenetic test to predict severe toxicity to fluoropyrimidines.. Int J Cancer.

[17] Deenen MJ, Meulendijks D, Cats A, Sechterberger MK, Severens JL (2016). Upfront Genotyping of DPYD*2A to individualize fluoropyrimidine therapy: a safety and cost analysis.. J Clin Oncol.

[18] Henricks LM, Lunenburg CATC, Meulendijks D, Gelderblom H, Cats A (2015). Translating DPYD genotype into DPD phenotype: using the DPYD gene activity score.. Pharmacogenomics.

[19] Amstutz U, Henricks LM, Offer SM, Barbarino J, Schellens JHM (2018). Clinical pharmacogenetics implementation consortium (CPIC) guideline for dihydropyrimidine dehydrogenase genotype and fluoropyrimidine dosing: 2017 update.. Clin Pharmacol Ther.

[20] Lunenburg CATC, Henricks LM, Dreussi E, Peters FP, Fiocco M (2018). Standard fluoropyrimidine dosages in chemoradiation therapy result in an increased risk of severe toxicity in DPYD variant allele carriers.. Eur J Cancer.

[21] Meulendijks D, Henricks LM, Sonke GS, Deenen MJ, Froehlich TK (2015). Clinical relevance of DPYD variants c.1679T>G, c.1236G>A/HapB3, and c.1601G>A as predictors of severe fluoropyrimidine-associated toxicity: a systematic review and meta-analysis of individual patient data.. Lancet Oncol.

[22] Lunenburg CA, van Staveren MC, Gelderblom H, Guchelaar HJ, Swen JJ (2016). Evaluation of clinical implementation of prospective DPYD genotyping in 5-fluorouracil- or capecitabine-treated patients.. Pharmacogenomics.

[23] Innocenti F (2014). DPYD variants to predict 5-FU toxicity: the ultimate proof.. J Natl Cancer Inst.

[24] Roncato R, Cecchin E, Montico M, De Mattia E, Giodini L (2017). Cost evaluation of irinotecan-related toxicities associated with the UGT1A1*28 patient genotype.. Clin Pharmacol Ther.

[25] Toffoli G, Innocenti F, Polesel J, De Mattia E, Sartor F (2018). The genotype for DPYD risk variants in colorectal cancer patients and the related toxicity management costs in clinical practice.. Clin Pharmacol Ther.

[26] Henricks LM, Lunenburg CATC, de Man FM, Meulendijks D, Frederix GWJ (2019). A cost analysis of upfront DPYD genotype-guided dose individualisation in fluoropyrimidine-based anticancer therapy.. Eur J Cancer.

[27] Keeling NJ, Rosenthal MM, West-Strum D, Patel AS, Haidar CE (2017). Preemptive pharmacogenetic testing: exploring the knowledge and perspectives of US payers.. Genet Med..

[28] Cecchin E, Roncato R, Guchelaar HJ, Toffoli G, Ubiquitous Pharmacogenomics Consortium (2017). Ubiquitous pharmacogenomics (U-PGx): the time for implementation is now. An Horizon2020 Program to drive pharmacogenomics into clinical practice.. Curr Pharm Biotechnol.

[29] van der Wouden CH, Cambon-Thomsen A, Cecchin E, Cheung KC, Dávila-Fajardo CL (2017). Implementing pharmacogenomics in Europe: design and implementation strategy of the ubiquitous pharmacogenomics consortium.. Clin Pharmacol Ther.

[30] Duthie SJ (2011). Folate and cancer: how DNA damage, repair and methylation impact on colon carcinogenesis.. J Inherit Metab Dis.

[31] De Mattia E, Toffoli G (2009). C677T and A1298C MTHFR polymorphisms, a challenge for antifolate and fluoropyrimidine-based therapy personalisation.. Eur J Cancer.

[32] Toffoli G, Rossi D, Gaidano G, Cecchin E, Boiocchi M (2003). Methylenetetrahydrofolate reductase genotype in diffuse large B-cell lymphomas with and without hypermethylation of the DNA repair gene O6-methylguanine DNA methyltransferase.. Int J Biol Markers.

[33] Boisdron-Celle M, Capitain O, Faroux R, Borg C, Metges JP (2017). Prevention of 5-fluorouracil-induced early severe toxicity by pre-therapeutic dihydropyrimidine dehydrogenase deficiency screening: assessment of a multiparametric approach.. Semin Oncol.

[34] Terrazzino S, Cargnin S, Del Re M, Danesi R, Canonico PL (2013). DPYD IVS14+1G>A and 2846A>T genotyping for the prediction of severe fluoropyrimidine-related toxicity: a meta-analysis.. Pharmacogenomics.

[35] Falvella FS, Cheli S, Martinetti A, Mazzali C, Iacovelli R (2015). DPD and UGT1A1 deficiency in colorectalcancerpatientsreceivingtriple tchemotherapy with fluoropyrimidines, oxaliplatin and irinotecan.. Br J Clin Pharmacol.

[36] Madi A, Fisher D, Maughan TS, Colley JP, Meade AM (2018). Pharmacogenetic analyses of 2183 patients with advanced colorectal cancer; potential role for common dihydropyrimidine dehydrogenase variants in toxicity to chemotherapy.. Eur J Cancer.

[37] Pellicer M, García-González X, García MI, Blanco C, García-Alfonso P (2017). Use of exome sequencing to determine the full profile of genetic variants in the fluoropyrimidine pathway in colorectal cancer patients affected by severe toxicity.. Pharmacogenomics.

[38] Ruzzo A, Graziano F, Galli F, Galli F, Rulli E (2017). Dihydropyrimidinedehydrogenase pharmacogenetics for predicting fluoropyrimidine-related toxicity in the randomised, phase III adjuvant TOSCA trial in high-risk colon cancer patients.. Br J Cancer.

[39] Rosmarin D, Palles C, Church D, Domingo E, Jones A (2014). Genetic markers of toxicity from capecitabine and other fluorouracil-based regimens: investigation in the QUASAR2 study, systematic review, and meta-analysis.. J Clin Oncol.

[40] Offer SM, Wegner NJ, Fossum C, Wang K, Diasio RB (2013). Phenotypic profiling of DPYD variations relevant to 5-fluorouracil sensitivity using real-time cellular analysis and in vitro measurement of enzyme activity.. Cancer Res.

[41] He YF, Wei W, Zhang X, Li YH, Li S (2008). Analysis of the DPYD gene implicated in 5-fluorouracil catabolism in Chinese cancer patients.. J Clin Pharm Ther.

[42] Offer SM, Fossum CC, Wegner NJ, Stuflesser AJ, Butterfield GL (2014). Comparative functional analysis of DPYD variants of potential clinical relevance to dihydropyrimidine dehydrogenase activity.. Cancer Res.

[43] Nie Q, Shrestha S, Tapper EE, Trogstad-Isaacson CS, Bouchonville KJ (2017). Quantitative contribution of rs75017182 to dihydropyrimidine dehydrogenase mRNA splicing and enzyme activity.. Clin Pharmacol Ther.

[44] Launay M, Dahan L, Duval M, Rodallec A, Milano G (2016). Beating the odds: efficacy and toxicity of dihydropyrimidine dehydrogenase-driven adaptive dosing of 5-FU in patients with digestive cancer.. Br J Clin Pharmacol.

[45] Launay M, Ciccolini J, Fournel C, Blanquicett C, Dupuis C (2017). Upfront DPD deficiency detection to secure 5-FU administration: part 2-application to head-and-neck cancer patients.. Clin Cancer Drugs.

[46] Meulendijks D, Henricks LM, Jacobs BAW, Aliev A, Deenen MJ (2017). Pretreatment serum uracil concentration as a predictor of severe and fatal fluoropyrimidine-associated toxicity.. Br J Cancer.

[47] Etienne-Grimaldi MC, Boyer JC, Beroud C, Mbatchi L, van Kuilenburg A (2017). New advances in DPYD genotype and risk of severe toxicity under capecitabine.. PLoS One.

[48] Botticelli A, Onesti CE, Strigari L, Occhipinti M, Di Pietro FR (2017). A nomogram to predict 5-fluorouracil toxicity: whenpharmacogenomicsmeets the patient.. Anticancer Drugs.

[49] Meulendijks D, Rozeman EA, Cats A, Sikorska K, Joerger M (2017). Pharmacogenetic variants associated with outcome in patients with advanced gastric cancer treated with fluoropyrimidine and platinum-based triplet combinations: a pooled analysis of three prospective studies.. Pharmacogenomics J.

[50] Cecchin E, Russo A, Campagnutta E, Martella L, Toffoli G (2004). Lack of association of CYP1 B1*3 polymorphism and ovarian cancer in a Caucasian population.. Int J Biol Markers.

[51] Longley DB, Harkin DP, Johnston PG (2003). 5-fluorouracil: mechanisms of action and clinical strategies.. Nat Rev Cancer.

[52] De Mattia E, Cecchin E, Toffoli G (2015). Pharmacogenomics of intrinsic and acquired pharmacoresistance in colorectal cancer: toward targeted personalized therapy.. Drug Resist Updat.

[53] Lima A, Azevedo R, Sousa H, Seabra V, Medeiros R (2013). Current approaches for TYMS polymorphisms and their importance in molecular epidemiology and pharmacogenetics.. Pharmacogenomics.

[54] Jennings BA, Kwok CS, Willis G, Matthews V, Wawruch P (2012). Functional polymorphisms of folate metabolism and response to chemotherapy for colorectal cancer, a systematic review and meta-analysis.. Pharmacogenet Genomics.

[55] Campbell JM, Stephenson MD, Bateman E, Peters MDJ, Keefe DM (2017). Irinotecan-induced toxicity pharmacogenetics: an umbrella review of systematic reviews and meta-analyses.. Pharmacogenomics J.

[56] Smyth E, Zhang S, Cunningham D, Wotherspoon A, Soong R (2017). Pharmacogenetic analysis of the UK MRC (Medical Research Council) MAGIC trial: association of polymorphisms with toxicity and survival in patients treated with perioperative epirubicin, cisplatin, and 5-fluorouracil (ECF) chemotherapy.. Clin Cancer Res.

[57] Tan BR, Thomas F, Myerson RJ, Zehnbauer B, Trinkaus K (2011). Thymidylate synthase genotype-directed neoadjuvant chemoradiation for patients with rectal adenocarcinoma.. J Clin Oncol.

[58] Rosmarin D, Palles C, Pagnamenta A, Kaur K, Pita G (2015). A candidate gene study of capecitabine-related toxicity in colorectal cancer identifies new toxicity variants at DPYD and a putative role for ENOSF1 rather than TYMS.. Gut.

[59] García-González X, Cortejoso L, García MI, García-Alfonso P, Robles L (2015). Variants in CDA and ABCB1 are predictors of capecitabine-related adverse reactions in colorectal cancer.. Oncotarget.

[60] Cecchin E, Agostini M, Pucciarelli S, De Paoli A, Canzonieri V (2011). Tumor response is predicted by patient genetic profile in rectal cancer patients treated with neo-adjuvant chemo-radiotherapy.. Pharmacogenomics J.

[61] Libra M, Navolanic PM, Talamini R, Cecchin E, Sartor F (2004). Thymidylate synthetase mRNA levels are increased in liver metastases of colorectal cancer patients resistant to fluoropyrimidine-based chemotherapy.. BMC Cancer.

[62] Cecchin E, Perrone G, Nobili S, Polesel J, De Mattia E (2015). MTHFR-1298 A>C (rs1801131) is a predictor of survival in two cohorts of stage II/III colorectal cancer patients treated with adjuvant fluoropyrimidine chemotherapy with or without oxaliplatin.. Pharmacogenomics J.

[63] Pellicer M, García-González X, García MI, Robles L, Grávalos C (2017). Identification of new SNPs associated with severe toxicity to capecitabine.. Pharmacol Res.

[64] Ose J, Botma A, Balavarca Y, Buck K, Scherer D (2018). Pathway analysis of genetic variants in folate-mediated one-carbon metabolism-related genes and survival in a prospectively followed cohort of colorectal cancer patients.. Cancer Med.

[65] Ichikawa W, Takahashi T, Suto K, Sasaki Y, Hirayama R (2006). Orotate phosphoribosyltransferase gene polymorphism predicts toxicity in patients treated with bolus 5-fluorouracil regimen.. Clin Cancer Res.

[66] Tsunoda A, Nakao K, Watanabe M, Matsui N, Ooyama A (2011). Associations of various gene polymorphisms with toxicity in colorectal cancer patients receiving oral uracil and tegafur plus leucovorin: a prospective study.. Ann Oncol.

[67] Gusella M, Bertolaso L, Bolzonella C, Pasini F, Padrini R (2011). Frequency of uridine monophosphate synthase Gly(213)Ala polymorphism in Caucasian gastrointestinal cancer patients and healthy subjects, investigated by means of new, rapid genotyping assays.. Genet Test Mol Biomarkers.

[68] Jennings BA, Loke YK, Skinner J, Keane M, Chu GS (2013). Evaluating predictive pharmacogenetic signatures of adverse events in colorectal cancer patients treated with fluoropyrimidines.. PLoS One.

[69] Loganayagam A, Arenas Hernandez M, Corrigan A, Fairbanks L, Lewis CM (2013). Pharmacogenetic variants in the DPYD, TYMS, CDA and MTHFR genes are clinically significant predictors of fluoropyrimidine toxicity.. Br J Cancer.

[70] Caronia D, Martin M, Sastre J, de la Torre J, García-Sáenz JA (2011). A polymorphism in the cytidine deaminase promoter predicts severe capecitabine-induced hand-foot syndrome.. Clin Cancer Res.

[71] Dahan L, Ciccolini J, Evrard A, Mbatchi L, Tibbitts J (2012). Sudden death related to toxicity in a patient on capecitabine and irinotecan plus bevacizumab intake: pharmacogenetic implications.. J Clin Oncol.

[72] Lévi F, Karaboué A, Saffroy R, Desterke C, Boige V (2017). Pharmacogenetic determinants of outcomes on triplet hepatic artery infusion and intravenous cetuximab for liver metastases from colorectal cancer (European trial OPTILIV, NCT00852228).. Br J Cancer.

[73] Yap YS, Kwok LL, Syn N, Chay WY, Chia JWK (2017). Predictors of hand-foot syndrome and pyridoxine for prevention of capecitabine-induced hand-foot syndrome: a randomized clinical trial.. JAMA Oncol.

[74] De Mattia E, Cecchin E, Montico M, Labriet A, Guillemette C (2018). Association of STAT-3 rs1053004 and VDR rs11574077 With FOLFIRI-related gastrointestinal toxicity in metastatic colorectal cancer patients.. Front Pharmacol.

[75] Garziera M, Virdone S, De Mattia E, Scarabel L, Cecchin E (2017). HLA-G 3’UTR polymorphisms predict drug-induced G3-4 toxicity related to folinic acid/5-fluorouracil/oxaliplatin (FOLFOX4) chemotherapy in non-metastatic colorectal cancer.. Int J Mol Sci.

[76] Garziera M, Bidoli E, Cecchin E, Mini E, Nobili S (2015). HLA-G 3’UTR polymorphisms impact the prognosis of stage II-III CRC patients in fluoropyrimidine-based treatment.. PLoS One.

[77] De Mattia E, Dreussi E, Montico M, Gagno S, Zanusso C (2018). A clinical-genetic score to identify surgically resected colorectal cancer patients benefiting from an adjuvant fluoropyrimidine-based therapy.. Front Pharmacol.

[78] Re VD, Caggiari L, Zorzi MD, Talamini R, Racanelli V (2014). Genetic diversity of the KIR/HLA system and outcome of patients with metastatic colorectal cancer treated with chemotherapy.. PLoS One.

[79] Abad A, Martínez-Balibrea E, Viéitez JM, Alonso-Orduña V, García Alfonso P (2018). Genotype-based selection of treatment of patients with advanced colorectal cancer (SETICC): a pharmacogenetic-based randomized phase II trial.. Ann Oncol.

[80] McLeod HL, Sargent DJ, Marsh S, Green EM, King CR (2010). Pharmacogenetic predictors of adverse events and response to chemotherapy in metastatic colorectal cancer: results from North American Gastrointestinal Intergroup Trial N9741.. J Clin Oncol.

[81] Toffoli G, Cecchin E, Gasparini G, D’Andrea M, Azzarello G (2010). Genotype-driven phase I study of irinotecan administered in combination with fluorouracil/leucovorin in patients with metastatic colorectal cancer.. J Clin Oncol.

[82] Di Francia R, Frigeri F, Berretta M, Cecchin E, Orlando C (2010). Decision criteria for rational selection of homogeneous genotyping platforms for pharmacogenomics testing in clinical diagnostics.. Clin Chem Lab Med.

[83] Afzal S, Gusella M, Vainer B, Vogel UB, Andersen JT (2011). Combinations of polymorphisms in genes involved in the 5-Fluorouracil metabolism pathway are associated with gastrointestinal toxicity in chemotherapy-treated colorectal cancer patients.. Clin Cancer Res.

[84] Kozyra M, Ingelman-Sundberg M, Lauschke VM (2017). Rare genetic variants in cellular transporters, metabolic enzymes, and nuclear receptors can be important determinants of interindividual differences in drug response.. Genet Med.

